# Analysis of gene mutations involved in chloroquine resistance in *Plasmodium falciparum* parasites isolated from patients in the southwest of Saudi Arabia

**DOI:** 10.4103/0256-4947.62826

**Published:** 2010

**Authors:** Saad M. Bin Dajem, Ahmed Al-Qahtani

**Affiliations:** a From the Biology Department, College of Science, King Khalid University, Abha, Saudi Arabia; b From the Biological and Medical Research, King Faisal Specialist Hospital and Research Centre, Riyadh, Saudi Arabia

## Abstract

**BACKGROUND AND OBJECTIVES:**

Chloroquine has been the drug of choice for the treatment of malaria for many decades. We aimed to examine the molecular basis of chloroquine resistance among *Plasmodium falciparum* isolates from the southwestern region of Saudi Arabia by analyzing the K76T and N86Y mutations in the PfCRT and PfMDR1 genes, respectively.

**PATIENTS AND METHODS:**

*P falciparum*-infected blood spot samples (n=121) were collected on filter papers. DNA was extracted and fragments from the above genes were amplified using nested PCR. The amplicons were digested by ApoI enzyme and sequenced.

**RESULTS:**

Of the 121 samples, 95 and 112 samples could be amplified for PfCRT K76T and PfMDR1 N86Y mutations, respectively. All of the samples amplified for the PfCRT K76T mutation were undigestible by ApoI, suggesting the presence of the K76T mutation. For the PfMDR1 N86Y mutation, 65/109 samples (59.6%) were digestible when treated with ApoI in a pattern, suggestive of the presence of the investigated wild allele (N86). However, 44/109 samples (40.4%) were digestible by ApoI, suggesting the presence of the mutated allele (Y) at position 86. DNA sequencing confirmed these results.

**CONCLUSION:**

Surprisingly, all isolates exhibited the mutated allele at codon 76 (K76T) of PfCRT. However, the mutated mutant allele at codon 86 (N86Y) of PfMDR1 was found in 40.4% of the samples studied. To our knowledge, this is the first study that has investigated the existence of the mutation in the PfMDR1 gene in the country. This study will contribute to the development of new strategies for therapeutic intervention against malaria in Saudi Arabia.

Malaria is a serious global public health problem caused by the protozoan parasites belonging to the genus *Plasmodium*. Whereas *Plasmodium vivax, P malariae,* and *P ovale* give rise to considerable malaria morbidity, only *P falciparum* results in high mortality. The disease is considered to be re-emerging, largely due to the spread of malarial drug resistance, insecticide resistance, and the rise in population and travel.^1^ About 40% of the world's population lives in areas of malaria transmission in nearly 100 countries, and infection with malaria results in 1.1-2.7 million deaths annually.^2^,^3^

According to the World Health Organization Eastern Mediterranean Region (WHO-EMR) report, Saudi Arabia is considered an endemic country for malaria transmission,^4^ with most cases diagnosed in the Jazan and Aseer regions located in the southwest part of the country. Nearly 96% of the cases are caused by *P falciparum* with *Anopheles arabiensis* being the main vector for transmission.^5^–^8^

The malaria control program in Saudi Arabia is based mainly on integrated control measures, including indoor spraying of insecticides, case detection, and treatment. High rates of population movement of expatriates working in the country and pilgrims between Saudi Arabia and malaria-endemic countries, such as Sudan, India, and Yemen, complicate the control of the disease.^9^,^10^

Chloroquine is the drug of choice for the treatment of complicated malaria cases. However, another combination of two drugs (sulfadoxine and pyrimethamine [SP]) is also effective against the disease. The current mainstream view of chloroquine antimalarial action is that chloroquine kills malaria parasites by binding to its target, a nonprotein molecule, ferriprotoporphyrin IX in lysosomes (food vacuoles) to form a toxic complex that lyses the parasites.^11^ The emergence and spread of chloroquine resistance (CQR) has been a problem for world health.^12^ In Saudi Arabia, the strategy to treat malaria cases was changed in 2008 by the Saudi health authorities with the introduction of artemisinin combination therapy (ACT) that consists of SP and artemisinin.^13^ Saudi Arabia is one of the few areas where *P falciparum* remained sensitive to chloroquine until the early 1990s despite the fact that CQR emerged in malaria-endemic countries close to Saudi Arabia, such as Iran, Pakistan, and East Africa where CQR was first reported in the early 1980s.^14^,^15^ A recent study examined a limited number of samples from the Jazan region for *P falciparum* chloroquine-resistant transporter (PfCRT) gene and found a CQR rate of 89.5%.^16^

Over the years, the emergence of drug-resistant parasites has hampered the efforts to control malaria worldwide. Since the late 1950s, reports of CQR have been documented in all endemic areas. Mutations in two genes, namely, PfCRT and *P falciparum* multidrug-resistant gene 1 (PfMDR1) have been implicated in resistance to chloroquine.^17^ Several point mutations in the PfCRT gene have been shown to correlate with resistance. Of these, only the mutation of lysine to threonine at the 76th position (K76T) in CRT is significantly found in resistant strains of malaria from different endemic areas of the world.^18^,^19^ Other mutations observed in the PfMDR1 gene were found to be strongly linked to CQR in various regions.^20^ Several field studies have related CQR to the mutation of aspargine to tyrosine at the 86th position (N86Y) of the MDR1 protein.^21^,^22^ In this study, we examined the presence of K76T and N86Y mutations in PfCRT and PfMDR1, respectively, among *P falciparum* parasites isolated from the southwestern region of Saudi Arabia.

## PATIENTS AND METHODS

The cases studied in this report were individuals (n=121) suspected of malaria infection who visit the Malaria Center in Aseer and Jazan Provinces. Only malaria cases confirmed with blood microscopic examination were included in this study. Blood samples (nearly 50 μL) were collected by finger-pricking and three drops from each patient were blotted onto Whatman (3 mm) chromatography paper. The spotted blood samples were placed in plastic bags and transported to the laboratory. All patients who participated in this study signed a consent form and the study was approved by the Ethics Committee at King Khalid University, Abha, Saudi Arabia.

DNA was extracted from the spotted blood samples according to the method of Sakihama et al with some modifications.^23^ Briefly, filter papers containing blood spots were placed into 1.5 mL Eppendorf tubes and 0.5% saponin was added and incubated overnight at 4°C. One hundred microliters of 5% Chelex-100 solution (Bio-Rad Laboratories, USA) were added to each tube and incubated at 100°C for 5 minutes in a heating block with vortexing every one minute. The supernatant was transferred to a new tube and stored at 4°C until further processing.

The primers used in this study were originally based on published data.^24^ A set of outer and inner primers was synthesized for the amplification of DNA fragments from PfCRT and PfMDR1 genes. Primer sequences, expected molecular weight of amplicons, and the PCR conditions are illustrated in Table 1. For digestions, aliquots of PfCRT and PfMDR1 PCR products were digested with 10 U of the restriction enzyme ApoI (New England Laboratories, USA), according to the manufacturer's recommendations. All digested products were electrophoretically processed in 2% agarose gels and the bands were visualized with 1 mg/mL ethidium bromide under UV light. DNA sequencing was carried out at the DNA sequencing core facility at the Research Center of King Faisal Specialist Hospital and Research Center (KFSHRC), using BigDye Terminator v3.1 Cycle Sequencing Kit according to the manufacturer's instructions (BigDyeTerminator v3.1 Cycle Sequencing Kit, Applera, USA) and the sequences were analyzed using DNASTAR software.

**Table 1:** Primers, expected molecular weight of amplicons, and the PCR conditions for genes investigated in this study.

Primers		Sequence (5'–3')	Size (bp)	PCR conditions
**First round**				
PfMDR1	OMDR1/F	TGTTGAAAGATGGGTAAAGAGCAGAAAGAG	660	94°C, 3 min followed by 45 cycles (94°C, 30 sesc; 56 °C, 30 secs; 60°C, 30 secs); 60°C, 3 min
	OMDR1/R	TACTTTCTTATTACATATGACACCACAAAC		
PfCRT	OCRT/F	CCGTTAATAATAAATACACGCAC	1389	
	OCRT/R	CGGATGTTACAAAACTATAGTTAC		
**Second round**				
PfMDR1	IMDR1/F	GTCAAACGTGCATTTTTATTAATGACCATTTA	560	94°C, 3 min followed by 40 cycles (94°C, 30 secs; 47°C, 30 secs; 68°C, 1 min); 64°C, 3 mins
	IMDR1/R	AAAGATGGTAACCTCAGTATCAAAGAGAG		
PfCRT	ICRT/F	TGTGCTCATGTGTTTAAACTT	145	
	ICRT/R	CAAAACTATAGTTACCAATTTTG		

## RESULTS

### Analysis of the PfCRT K76T codon

**Figure 1:**
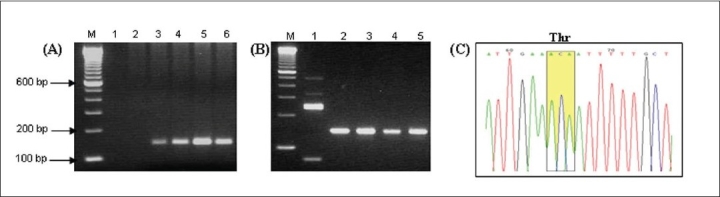
(A) Agarose gel of typical PCR amplification patterns of the nested PCR products of the DNA from P falciparum-infected blood samples for PFCRT gene, showing the expected product of 154 bp. M: 100-bp ladder, lanes 1 and 2: no-template negative controls, lanes 3-6: PCR product from representative patient samples. (B) Patterns obtained after restriction digestion of the PCR products with ApoI. Lane 1: restriction digestion control, lanes 2-5: PCR product from representative patient samples after digestion with ApoI. (C) Chromatogram confirming the presence of mutation at the 76th amino acid position highlighted in yellow

Of the 121 samples examined, 26 (21.5%) were not amplifiable but the remaining 95 samples (78.5%) gave amplification products (Table 2) and the amplicons of expected molecular weight (145 bp) were visualized after nested PCR (Figure 1a). None of the 95 amplified samples (100%) could be digested by ApoI, suggesting the presence of the mutation of interest (76T) that indicates chloroquine resistance (Figure 1b). DNA sequencing of the PCR products of these samples confirmed the results obtained by the PCR/RFLP method (Figure 1c).

**Table 2:** Prevalence of wild and mutant alleles of PfCRT and PfMDR1 conferring resistance to chloroquine in *P falciparum* isolates from Southwest parts of Saudi Arabia.

Gene	N	Wild Allele	Mutant Allele	Mutation (%)
PfCRT	95	76K (0/95)	76T (95/95)	100
PfMDR1	109	86N (65/109)	86Y (44/109)	40.4

### Analysis of the PfMDR1 N86Y codon

**Figure 2:**
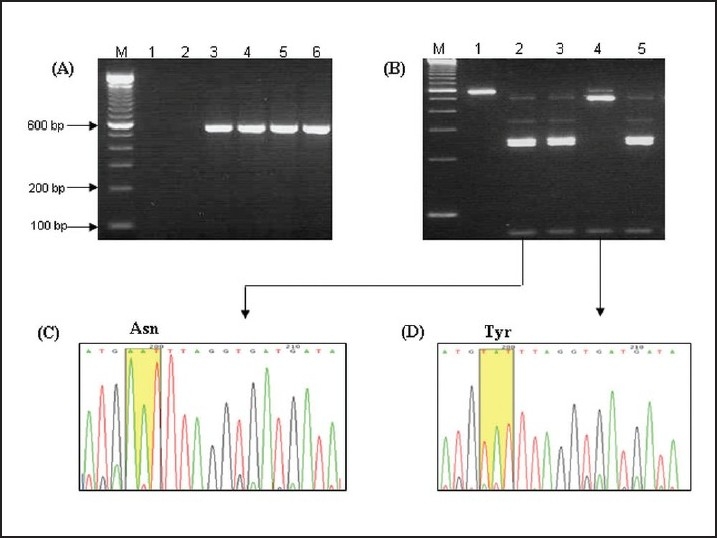
(A) Agarose gel of typical PCR amplification patterns of the nested PCR products of the DNA from *P falciparum*-infected blood samples for PFMDR1 gene, giving the expected product of 560 bp. M: 100-bp ladder, lanes 1 and 2: no-template negative controls, lanes 3-6: PCR product from representative patient samples. (B) Patterns obtained after restriction digestion of the PCR products with ApoI. The products of the wild-type allele are 250 bp, 231 bp and 79 bp, while, two fragments of 481 bp and 97 bp are evident for the mutant alleles. M: 100-bp ladder, lanes 1: undigested PCR product, lanes 2-5: PCR product from representative patient samples after digestion with ApoI. (C and D) representative chromatograms confirming the presence of mutant and wild alleles at the 86th amino acid position.

Of the 121 samples analyzed, 112 samples (92.5%) yielded amplification products (Table 2) and the amplicons of expected molecular weight (560 bp) were visualized after nested PCR (Figure 2a). Of the total samples examined, 9 (7.5%) were not amplifiable under the conditions used in this study. This suggested that these samples could have been misdiagnosed for malaria or that it could have been a form of malaria caused by a species other than *P falciparum*. Of the 109 correctly amplified samples, 65 (59.6%) gave 250, 231, and 79 bp bands after ApoI digestion, indicating the presence of the wild-type 86N codon. On the other hand, 44 (40.4%) samples gave 481 and 97 bp bands after ApoI digestion, indicating the presence of the mutated 86Y codon (Figure 2b). The remaining three (of the 112 yielding amplification products) samples showed faint bands that were not appropriate for the digestion assay. DNA sequencing confirmed the results obtained by PCR/RFLP analysis (Figure 2c and 2d).

## DISCUSSION

At present, there is little information on the association of genetic variation in *P falciparum* parasites with drug resistance in Saudi Arabia. As many field and laboratory studies have investigated the association of CQR with specific mutations in codon 76 of the PfCRT gene and at codon 86 of the PfMDR1 gene, we aimed to investigate these mutations by PCR-RFLP and DNA sequencing technologies in isolates from the southwestern part of the country. Chloroquine was introduced in the 1940s as the drug of choice for malaria treatment.^25^ However, parasite resistance to chloroquine appeared after more than 60 years of intensive use. Extended parasite exposure to chloroquine has necessitated the exploration of the characteristics of *P falciparum* population genetics in relation to resistance to chloroquine.

We used standard molecular biology methods to study mutations linked to resistance in PfCRT and PfMDR genes.^24^ However, not all samples were amplifiable as only 95 and 112 samples were amplified for the PfCRT K76T and PfMDR1 N86Y mutations, respectively. Such problems have been documented with different PCR conditions. For example, Sutherland et al were able to amplify PfCRT in 98/108 (90.7%) samples and to amplify PfMDR1 in 74/108 samples (68.5%).^26^

When the PfCRT gene was identified, many studies confirmed the presence of the K76T mutation in CQR parasites.^27^ The investigation of another gene, PfMDR1,^28^ revealed that a mutation at the 86th position was strongly linked to CQR in laboratory clones, even though it is not the sole requirement for CQR.^29^ Globally, it has been found that replacement of the lysine (a positively charged amino acid) in PfCRT with threonine (an uncharged amino acid) at position 76 confers resistance to chloroquine.^30^ The very high prevalence of the PfCRT 76T variant in our study is in agreement with the findings of other studies where clinical chloroquine treatment failure was evident.^31^ A study from Iran reported that PfCRT 76T was found in 99% of the investigated samples.^32^ In a recent prevalence study reported from Thailand, the PfCRT 76T allele frequency was found in 99.1% of the investigated samples.^33^ A molecular prevalence survey has shown that the prevalence of the PfCRT 76T mutation was over 90% in Yunnan province, China.^34^ A study of PfCRT point mutations and the level of CQR in *P falciparum* isolates imported into Europe from Congo and Tanzania showed that the frequency of the 76T mutated allele was 71.4%.^35^ The presence of the PfCRT K76T mutation from Papua New Guinea was found to be 92.9%.^36^ A recent study from the Philippines describing *P falciparum* isolates from three areas of the country showed that the frequency of the PfCRT 76T mutation was found to be 100% in Kalinga, 80% in Palawan, and 87% in Mindanao.^37^

Different PfCRT haplotypes have been reported from malarial isolates in endemic areas. The CQR-associated haplotype (amino acids from 72 to 76) detected in this study was the CVIET. This is considered the typical haplotype isolated from Southeast Asia and the African continent.^38^ Finding the same haplotypes in Saudi Arabia could be attributed to the continuous travel and human migration related to employment, tourism, or religious pilgrimage.^39^,^40^

Currently, mutations detected in PfMDR1 have been hypothesized to augment the level of resistance in CQR *P falciparum* parasites.^24^,^41^ In our study, the PfMDR1 86Y allele frequencies were found to be 59.6% and 40.4% for the N86 allele. A similar finding was reported from Iran where it was found that PfMDR1 86Y appeared in 72% of the samples in the Sistan-Baluchistan Province.^31^ In Thailand, the PfMDR1 86N and 86Y alleles were identified in 75.5% and 20% of samples, respectively.^32^ Isolates from three distinct areas of the Philippines showed that the frequency of the PfMDR1 N86Y mutation was 39% in Kalinga town, 35% in Palawan town, and 93% in Mindanao town isolates.^36^ The PfMDR1 N86Y mutation was found in 23.1% of the isolates from Southeast Iran,^42^ which is a frequency pattern similar to that found in African regions where CQR is well established.^43^ Babiker and colleagues reported that the PfMDR1 N86Y mutation contributes more to CQR in the PfCRT 76T background in Sudanese isolates of *P falciparum*.^44^ Our PCR-RFLP analysis consistently showed incomplete digestion of the PCR product, which is suggestive of multiclonal infection of the same parasites.^45^ The molecular markers for CQR, when optimized and validated as tools for surveillance, will help in malaria control, guide national malaria treatment policies, and monitor changes in parasite drug susceptibility following changes in malaria drug treatment policy.
